# Onchocerciasis: shifting the target from control to elimination requires a new first-step—elimination mapping

**DOI:** 10.1093/inthealth/ihx052

**Published:** 2018-02-19

**Authors:** Maria P Rebollo, Honorat Zoure, Kisito Ogoussan, Yao Sodahlon, Eric A Ottesen, Paul T Cantey

**Affiliations:** 1 Expanded Special Project for Elimination of NTDs, Decatur, GA, USA; 2 Neglected Tropical Diseases Support Center, Task Force for Global Health, Decatur, Georgia, USA; 3 Mectizan Donation Program, Task Force for Global Health, Decatur, Georgia, USA; 4 World Health Organization, Geneva, Switzerland

**Keywords:** Elimination, ESPEN, Mapping, MDA, Onchocerciasis

## Abstract

The meaning of ‘mapping’ in relation to onchocerciasis has changed at least three times over the past 50 years as the programmatic goals and the assessment tools have changed. With the current goal being global elimination of *Onchocerca volvulus* (OV), all areas where OV might currently be transmitted and where mass drug administration (MDA) with ivermectin treatment has not been delivered previously must now be identified by careful, detailed ‘elimination mapping’ as either OV endemic or not, so that appropriate programmatic targets can be established. New tools and strategies for such elimination mapping have become available, though ongoing studies must still be completed to define agreed upon optimal diagnostic evaluation units, sampling strategies and serologic tools. With detailed guidance and technical support from the World Health Organization and with implementation and financial support from their global partners, the OV-endemic countries of Africa can soon complete their elimination mapping and then continue with MDA programmes to progressively achieve the same success in OV elimination as that already achieved by the growing list of formerly OV-endemic countries in the Americas.

## Introduction

In 1993 the International Task Force for Disease Eradication (ITFDE) identified six diseases it considered eradicable or potentially eradicable.^1^ Onchocerciasis was not one of these diseases, even though the closely related filarial disease lymphatic filariasis (LF) was. Why was it that in 1993 LF was considered by the ITFDE likely able to be eliminated but onchocerciasis was not?

For any disease to be recognized as eradicable (or eliminable^1^) there are two attributes that are essential. Both relate not so much to the biology of the disease as to the availability of specific tools to target it. First, there must be an effective method capable of detecting the infection. Second, there must be an effective intervention tool able to get rid of it. When the ITFDE evaluated LF and onchocerciasis in 1993, it was clear that both had similarly effective intervention tools (i.e., microfilaricidal drug regimens) that permitted targeting the microfilariae in blood (LF) or skin (onchocerciasis) that transmit infection to the vector mosquitoes or blackflies and give rise to the infective larvae able to transmit infection to people during blood meals. The strategy for using these drug treatment tools was also similar; namely, bringing the microfilaria prevalence in endemic communities down below a threshold (R_0_) where transmission is no longer sustainable. Admittedly, the diagnostic tool available for LF (an antigen-detection card test [ICT]) was more effective than the clinical assessments or skin-snip microscopy used to detect onchocerciasis, but there was another important difference between the two infections at that time as well—there was an evidence base for the feasibility of using these tools for LF elimination in areas of China, Brazil and several other locales.

Actually, onchocerciasis too had been eliminated in certain areas, but only by using very different tools and strategies—targeting not the infected people, but the vector blackflies. Indeed, the earlier (1974–2002) Onchocerciasis Control Program (OCP) in West Africa had very successfully used helicopters, fixed-wing aircraft and other tools in 11 West African countries to disperse pesticides in massive vector control efforts to eliminate onchocerciasis.^2,3^ While the OCP was recognized as being highly successful in limiting the spread of infection (and even eliminating it in some areas), it was acknowledged that both the cost of vector elimination and, in particular, the different ecology of *Onchocerca volvulus* in other endemic areas of Africa and the Americas made this blackfly-focused intervention strategy unfeasible on a global scale.^4^

## Control—the original paradigm for onchocerciasis programmes

For the programme that succeeded the OCP—the African Programme for Onchocerciasis Control (APOC; 1996–2015)—the goal remained not elimination of onchocerciasis but control of the infection to the point where significant disease of the eye and skin was no longer a public health problem.^5^ The strategy to achieve this control target relied on the recently available antiparasitic ivermectin, given as a single dose once a year through mass drug administration (MDA) to all populations at risk of significant disease. Additionally, because so many of the affected communities were in the very least accessible and most underserved regions, to reach these remote, endemic communities APOC created a novel health delivery strategy—Community Directed Treatment with Ivermectin (CDTI)—which has now been widely used and with great success both for onchocerciasis programmes and other health interventions.^6–8^

Most importantly, because the global goal for onchocerciasis was control (not elimination), determining where to initiate MDA (through CDTI) depended on where clinical eye and skin disease was most significant,^9^ and since onchocerciasis is most prominent in highly endemic areas, it was those areas that had to be defined by ‘mapping’ for preferential targeting through MDA.

## Elimination—the new paradigm for onchocerciasis programmes

In 2002, despite the established consensus that control was the appropriate global health target for onchocerciasis, the ITFDE and the World Health Organization (WHO) convened a large meeting of experts to review once again the feasibility of onchocerciasis elimination. Indeed, since the 1993 ITFDE assessment 9 years earlier there had been not only the development and availability of a new, sensitive onchocerciasis diagnostic tool (OV16 serology), but also much greater experience with the onchocerciasis intervention tool—the use and effective delivery through MDA of repeated, single-dose ivermectin to even the most remote at-risk communities. While there was agreement at that meeting that the same general strategy used for the elimination of LF as a public health problem—namely, MDA targeting all at-risk populations with single-dose microfilaricidal treatment once yearly for the duration of the reproductive life span of the adult parasites—would also be effective for onchocerciasis, the epidemiologic feasibility of effectively targeting and reaching all at-risk populations in Africa with the available diagnostic and intervention tools still seemed too daunting to change the target from control to elimination. For the Americas, however, the conclusion was different. As the burden of infection was very much less, the foci of infection more restricted, the at-risk populations for the most part more readily accessible and the political support in affected countries quite substantial, onchocerciasis was felt to be eliminable from the Americas.^10^

The final step in the paradigm shift from onchocerciasis control to elimination as the global target was stimulated by two sets of observations. The first was the progressive success of the onchocerciasis elimination efforts in the Americas,^11^ using the tools already available for diagnosis (OV16 serology in humans and parasite detection by polymerase chain reaction in vectors) and intervention (MDA with ivermectin). Indeed, in 11 of the 13 originally endemic foci in the Americas, onchocerciasis has now been eliminated.^12–15^ The second was the recognition that similar success in interrupting transmission was occurring in multiple foci in Africa (including in Senegal, Mali, Uganda and Sudan), where effective MDA-based control programmes that had been ongoing for 15–17 years were found to have been successful in interrupting transmission.^16–20^ These two sets of observations added an important evidence base that had been missing in the earlier ITFDE assessments of 1993 and 2002. With this new evidence, the WHO recognized in its ‘roadmap’ for targeting the neglected tropical diseases (NTDs) that elimination is the appropriate target for onchocerciasis programmes not just in the Americas, but in Africa as well^21^—coordinated now in Africa through guidance from the WHO’s Regional Office (AFRO) and its new Expanded Special Project for Elimination of Neglected Tropical Diseases (ESPEN).

## Elimination mapping what is it and why is it necessary now?

As the goals for onchocerciasis programmes have evolved, the requirements for mapping have changed as well.

1. For the OCP (1974–2002), the target was interruption of transmission through blackfly control. Therefore, the mapping that was needed was definition of the breeding sites for the blackfly vectors being targeted with pesticides.
2. For the APOC (1996–2015), and for much of the effort in the second half of the OCP after ivermectin became available, the programme target was to prevent severe clinical eye and skin disease by ensuring sustainable delivery of ivermectin to at-risk populations. Therefore, the mapping needed was to define those areas in onchocerciasis-endemic countries of Africa where the prevalence of infection was above a threshold associated with severe eye disease so that these areas could be treated with ivermectin. That threshold was defined as a 20% prevalence of adult men in a community having nodules identified clinically by palpation (the REMO [Rapid Epidemiological Mapping for Onchocerciasis] strategy) or 35% prevalence when determined by skin microfilariae assessed microscopically in skin snips.^9,22^ Populations with findings below these thresholds were given the descriptor of being onchocerciasis hypo-endemic and they were felt not to require treatment since severe disease was generally not seen at those levels.
3. The shift of onchocerciasis programme targets to elimination requires an entirely new dimension in understanding the geographic distribution of infection. Elimination mapping, whose purpose is to determine exactly where additional treatment with ivermectin is required, must now identify all places that are not currently under treatment with ivermectin (for onchocerciasis or LF) and where the prevalence of *O. volvulus* infection is high enough to sustain transmission so that appropriate intervention can be provided. What this means, in practical terms, is that all those areas previously excluded from onchocerciasis control programmes because they had been defined as hypo-endemic or assumed to be non-endemic must now be reassessed to determine whether or not onchocerciasis is endemic at a level above the threshold where ongoing transmission is possible. Complicating this challenge, unfortunately, is the fact that neither this threshold nor the appropriate sampling strategy to define it has yet been determined.

## Critical challenges in formulating the steps in onchocerciasis elimination mapping

1. *Defining the operational unit for elimination mapping*. Knowing the biology of onchocerciasis and its vectors has for years led to thinking about this infection most naturally in terms of its transmission zones or foci; however, the more expedient operational unit for programmes is an administrative one, usually the district or equivalent. At the local level there is a great deal of knowledge that can identify where morbidity occurs, but it is virtually impossible without specific surveys to identify areas where ongoing transmission is present when morbidity is absent or quite low. Such areas, if left unidentified, could represent a significant threat for subsequent reintroduction of the disease to neighbouring treated areas. What this means is that while not all parts of a district are equal in terms of disease transmission or treatment requirements, for the purpose of defining the implementation needs of a country each district should be considered independently, identifying those districts that are completely free of disease and those that require treatment to interrupt transmission. Importantly, if the district-level assessment identifies portions of the district (subdistricts) where treatment decisions will differ from the rest of the district, the ‘implementation unit’ for treatment may be defined at a subdistrict level. While it is likely that in many instances the decision to implement would be uniform for any one district, only additional experience from assessing the currently unmapped hypo-endemic areas will settle this uncertainty.
2. *Agreeing on the tools and strategies to define a district with onchocerciasis*. The accuracy of identifying onchocerciasis-endemic areas needs to be much greater for elimination mapping than the mapping required for earlier control programmes, because all areas with any possibility of transmission must be identified. This means that the diagnostic tool must be optimized, the target population must be clearly identified and an effective sampling strategy must be defined.
  The earlier diagnostic tools for vector control of OV breeding sites or for disease control through clinical evaluation of nodules and microscopic detection of microfilariae in skin snips sufficed for previous mapping needs. However while positive findings using these tools can still be considered definitive, none of these diagnostics is sensitive enough to detect the lowest levels of infection still able to support onchocerciasis transmission.^14,23,24^ Fortunately, the availability of OV16 serology (either in enzyme-linked immunosorbent assay [ELISA] or rapid diagnostic test [RDT] format) provides a much more sensitive indicator of infection or exposure to infection^25,26^ since it detects a host’s antibody response to the parasite. At a cost of approximately $1.50 per test, it has become the community’s recommended replacement for older diagnostic tests. However, given the challenge of discriminating positive from negative responses with complete certainty, especially in populations having very low levels of infection, it is still necessary that measurable thresholds be established that take into account the specificity and sensitivity of the diagnostic. Therefore, for elimination mapping, national programmes expect the WHO to establish prevalence thresholds based on best evidence and consensus.
  The target populations sampled for the earlier mapping-for-control effort (REMO) were adult males, and, again, while positive findings in this group are considered definitive for identifying infection in the population, it is possible that a population more indicative of recent transmission would be children, the same group now recommended for assessment guiding the decision to stop MDA.^27^ For elimination mapping, national programmes expect WHO to establish a standardized target population for sampling based on best evidence and consensus.
  The earlier, ‘purposive’ sampling strategy to identify first- and second-line villages in relation to blackfly breeding sites along fast-flowing rivers remains valuable, but is not sufficient for onchocerciasis elimination mapping. In those countries where districts are considered the operational units for treatment, if purposive village sampling identifies onchocerciasis, those districts could be defined as endemic and treatment could start throughout. However, if the initial purposive village sampling does not detect onchocerciasis, further sampling is required—either additional purposive (in likely places) sampling or random cluster surveys (broadly across the district). In those countries where the entire district was not the agreed operational unit for treatment, random cluster surveys will have to be carried out in the entire district to define how broadly or narrowly treatment should be extended, even if the purposive sampling of villages has identified areas of ongoing transmission. Although local knowledge can and should guide decisions on where elimination mapping is needed, in areas where the vector is present and transmission is possible, random sampling will often be required. For elimination mapping, national programmes expect the WHO to establish a standardized sampling strategy based on best evidence and consensus.
3. *Adapting strategies to account for co-endemic filarial infections*. Complicating these uncertainties of diagnostic thresholds, target populations and sampling strategies for elimination mapping is the fact that such mapping must also take into account two other potentially co-endemic filarial infections (LF and *Loa loa* filariasis [loiasis]). Why? Because if LF is co-endemic, it is likely that LF elimination programmes are already under way or were carried out without prior definition of onchocerciasis prevalence, thus complicating the current understanding of onchocerciasis transmission, and if loiasis is co-endemic with onchocerciasis, there is the danger that using ivermectin to interrupt transmission of onchocerciasis might cause serious adverse events (including death) in individuals with very high levels of *L. loa* infection.^28^ Therefore, in such *Loa*-endemic settings, it is not enough to determine whether or not onchocerciasis is endemic; coordinated mapping for *L. loa* must also be undertaken to gauge the intensity of *L. loa* infection as efficiently as possible so that an appropriate, safe plan for MDA treatment can be designed. For elimination mapping to be carried out safely and efficiently, national programmes expect the WHO to establish strategies to address onchocerciasis co-endemicity with LF and/or loiasis based on best evidence and consensus.

## Getting started with elimination mapping

There really is no ‘getting started’ with elimination mapping, since all onchocerciasis mapping done to date is the first step of an elimination mapping effort whose current goal must be to focus on filling in those regions in onchocerciasis-endemic countries that were previously either unmapped or defined as hypo-endemic so that one can identify exactly where additional ivermectin treatment must now be initiated to eliminate onchocerciasis.

To understand how much onchocerciasis mapping remains to be done in each country, the necessary first step is to characterize each district (or subdistrict, if the operational treatment unit is not the district) in every onchocerciasis-endemic country into the following three epidemiologic categories, since the intervention approach to each will be different: ivermectin naïve and onchocerciasis present (i.e., above the agreed transmission threshold), ivermectin naïve and onchocerciasis absent (or below the transmission threshold) or ivermectin treated, either now or previously. Furthermore, in the 10 loiasis co-endemic countries of Central and West Africa, the assessment of *L. loa* infection and intensity levels must also be made for each district.

To guide this categorization effort, a comprehensive, efficient approach to characterizing the necessary epidemiologic variables in each district that also accounts for the ongoing surveys of concurrent LF elimination programmes has recently been created as a programmatic decision tool (algorithm). This algorithm recognizes the diagnostic needs in each district and provides recommendations for both mapping and treatment strategies. Its feasibility as a practical tool is currently being tested, but its full potential will be realized only after those specific thresholds, sampling strategies and diagnostic alternatives described above are defined. Fortunately, many of these uncertainties will be resolved empirically as the new tools and presumptive thresholds are utilized and tested in the current elimination mapping efforts and in the operational research being carried out by the WHO and its global partners.

**Figure 1.**
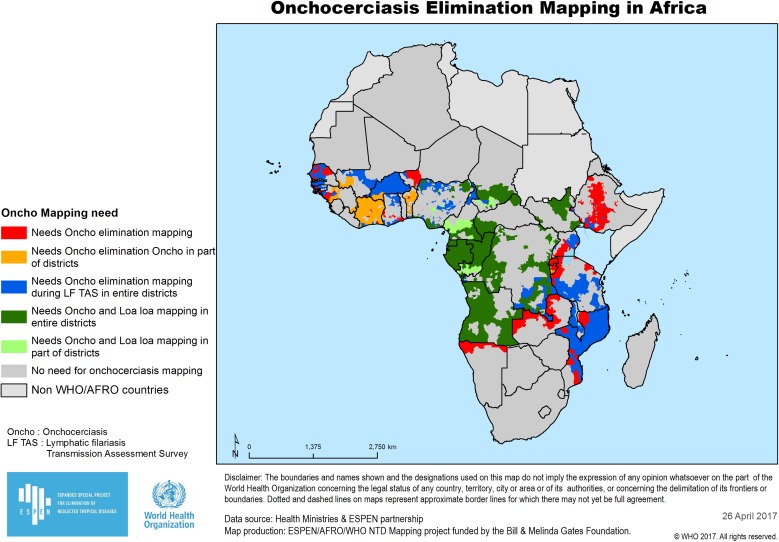
Elimination mapping needs. Distribution of districts in the WHO African Region that still need some additional assessment (specifics depending on information already available) in order to complete their elimination mapping. Data used to generate this pictorial display are taken from the AFRO NTD mapping data portal: http://ntd.afro.who.int/en/espen/home.

Already, the available onchocerciasis prevalence and treatment data collected earlier by the national programmes, by APOC and by the recent AFRO mapping initiative have been captured in a single database organized by district for all countries in the WHO’s Africa Region (http://ntd.afro.who.int/en/espen/home); these data are now in the process of confirmation and updating by national ministries of health. Although details in the data will certainly change after their local review and updating, the current information indicates that 2577 districts still need to be part of an elimination mapping effort. Of these, 786 are in *L. loa* co-endemic areas and the remaining 1791 are in non-*Loa* areas, 195 districts need only partial mapping because some areas of these district were previously treated with ivermectin during the APOC programmes and 713 are likely to be able to coordinate their elimination mapping with LF transmission assessment surveys being undertaken to evaluate the effectiveness of recently completed LF elimination programmes. While these data are most ‘actionable’ when visualized in line-item spreadsheets for each country and its districts, they also have been usefully displayed graphically (Figure 1).

## Immediate next steps

### At the international level

Most important at the international level is the recognition by everyone supporting these programmes that elimination mapping is essential to the ultimate success of OV elimination efforts. Elimination targets for onchocerciasis have been set for 2020,^21^ 2025^29^ and 2030,^30^ but none of these goals can be met if the extent of onchocerciasis infection remains uncertain.

Funding to initiate and then complete the onchocerciasis elimination mapping must be mobilized to support simultaneous mapping efforts and related operational research in all endemic countries of Africa. International agencies and bilateral and private sector donors will all be needed to supplement national funding sources, but such support will only be possible once the WHO and its international partners understand the costs of such mapping. Therefore, efforts to define these costs accurately are urgently needed. Again, there is no possibility for meeting onchocerciasis elimination targets without completing the necessary mapping, and there is not sufficient support to map without committed funding from engaged international and private sector donors.

The WHO will need to organize a meeting of experts to review the available evidence on the tools and strategies for elimination mapping in order to develop an interim way forward for mapping and to develop the key operational research questions that should be addressed.

### At the national level

Each country’s national programme needs to examine the available information for each of the districts that are both lacking data on onchocerciasis and ivermectin naïve in order to determine if any can be excluded from further assessment because of their epidemiologic unsuitability for onchocerciasis transmission. With such unsuitable districts excluded, the remaining districts can be included for mapping.

Once the WHO guidance on elimination mapping is available, countries can develop an implementation plan that will enable the roll-out of the mapping itself. This plan will include defining the target population for sampling through the purposive selection of first-line villages near known breeding sites within each district and the development of lists of communities or schools for undertaking the random selections to cover the entire district geographically. Operational and laboratory potential will also need to be evaluated to assess the national capacity to perform the surveys and ELISA (or RDT) testing. Where required, quality control, quality assurance and capacity enhancement will also need to be considered. The ESPEN (formerly APOC) Multi-Disease Surveillance Center laboratory in Ouagadougou can play a key role in providing for national laboratory support needs.

Training ministry of health teams to carry out the field work of elimination mapping must take place and can build on the appreciable experience gained during recent AFRO, APOC and ESPEN mapping initiatives targeting onchocerciasis and other NTDs. This training should be based on the WHO recommended algorithm, diagnostics and sampling strategies for both onchocerciasis and loiasis. It also must include the critical data capture and management component that is so essential for quality programmatic decision making. All of these programmatic elements—and their training materials—have been enhanced considerably in recent years and tools for disseminating them are now widely available.

## Conclusion

The concept of onchocerciasis mapping has changed at least three times over the past 50 years as the programmatic goals and assessment tools have changed. With the current goal of global elimination of onchocerciasis, all areas where onchocerciasis might be transmitted and where ivermectin treatment has not been delivered in the past must be defined as either onchocerciasis endemic or not by careful, detailed elimination mapping.

Fortunately, the tools and strategies for such elimination mapping are now available. With detailed guidance and technical support from the WHO and with technical and financial support from their global partners, the onchocerciasis-endemic countries of Africa can soon complete their elimination mapping challenges and continue with their MDA programmes to progressively achieve the same success in onchocerciasis elimination as the growing list of formerly onchocerciasis-endemic countries in the Americas have done.

## Footnotes

1 ‘Elimination’ can be defined as bringing *incident* infections to zero in a defined geographic area; ‘eradication’ is recognized when elimination has been achieved in *all* geographic areas.
